# From Phagocytes to Immune Defense: Roles for Coronin Proteins in *Dictyostelium* and Mammalian Immunity

**DOI:** 10.3389/fcimb.2018.00077

**Published:** 2018-03-22

**Authors:** Mayumi Mori, Ravindra Mode, Jean Pieters

**Affiliations:** Biozentrum, University of Basel, Basel, Switzerland

**Keywords:** coronins, *Dictyostelium discoideum*, Mycobacterium, phagocytes, immune cells

## Abstract

Microbes have interacted with eukaryotic cells for as long as they have been co-existing. While many of these interactions are beneficial for both the microbe as well as the eukaryotic cell, several microbes have evolved into pathogenic species. For some of these pathogens, host cell invasion results in irreparable damage and thus host cell destruction, whereas others use the host to avoid immune detection and elimination. One of the latter pathogens is *Mycobacterium tuberculosis*, arguably one of the most notorious pathogens on earth. In mammalian macrophages, *M. tuberculosis* manages to survive within infected macrophages by avoiding intracellular degradation in lysosomes using a number of different strategies. One of these is based on the recruitment and phagosomal retention of the host protein coronin 1, that is a member of the coronin protein family and a mammalian homolog of coronin A, a protein identified in *Dictyostelium*. Besides mediating mycobacterial survival in macrophages, coronin 1 is also an important regulator of naïve T cell homeostasis. How, exactly, coronin 1 mediates its activity in immune cells remains unclear. While in lower eukaryotes coronins are involved in cytoskeletal regulation, the functions of the seven coronin members in mammals are less clear. *Dictyostelium* coronins may have maintained multiple functions, whereas the mammalian coronins may have evolved from regulators of the cytoskeleton to modulators of signal transduction. In this minireview, we will discuss the different studies that have contributed to understand the molecular and cellular functions of coronin proteins in mammals and *Dictyostelium*.

## Introduction

All eukaryotes are surrounded by microorganisms that on the one hand fulfill important roles in providing symbiotic support for eukaryotic life, and at the same time can pose a threat in the form of virulent bacteria, causing infections, disease and death.

For both lower eukaryotes such as the amoeba *Dictyostelium discoideum*, as well as mammalian macrophages, the first encounter with microbes, especially bacteria, results in the activation of phagocytic processes leading to engulfment of the bacteria within phagosomes, followed by lysosomal digestion (Flannagan et al., 2012). When bacteria serve as nutrients, as is the case for *Dictyostelium*, lysosomal degradation will allow the availability of amino acids, lipids, and other molecules that serve the need for *Dictyostelium* growth (Allen and Aderem, 1996). In case of bacteria being phagocytosed by mammalian cells such as dendritic cells and macrophages, the end point is usually inactivation of the pathogen, as well as presentation of pathogen fragments to the immune system in order to activate effector lymphocytes (T cells) for the generation of adaptive immune responses (Pieters, 2000; Blum et al., 2013).

Phagocytosis is an ancient and evolutionary conserved mechanism (Aderem and Underhill, 1999). Indeed, the basic mechanisms regulating the formation of a phagocytic cup, internalization of the bacteria and the transfer from phagosomes to lysosomes are conserved from lower eukaryotes such as *Dictyostelium* to mammalian phagocytes, including macrophages, neutrophils and dendritic cells. Phagocytosis involves cell surface recognition through different plasma membrane receptors that transmit signals through a variety of pathways to the cytoskeleton in order to allow plasma membrane deformation to accommodate the incoming particles/bacteria (Flannagan et al., 2012).

### Virulence strategies employed by pathogenic microbes

Whereas the encounter of mammalian phagocytes with bacteria most often results in its destruction and activation of specific immunity, many pathogenic bacteria have evolved a diverse array of strategies to circumvent phagocytosis and lysosomal destruction. For example, several pathogens, including *Neisseria* spp., *Pseudomonas* spp., *Streptococcus* spp., and *Salmonella* spp. are known to avoid phagocytic uptake (Sarantis and Grinstein, 2012). Also, certain pathogens, including *Listeria* spp. and *Shigella* spp. are engulfed by phagocytic cells but rapidly transfer to the cytosol where they can proliferate and spread (Hamon et al., 2012; Mellouk and Enninga, 2016). Yet other bacilli such as *Brucella* spp. and *Salmonella* spp. enter the phagocyte through phagocytosis but divert to the endoplasmic reticulum (*Brucella* spp.) or the Golgi (*Salmonella* spp.) instead of being delivered to lysosomes (Celli and Gorvel, 2004; Escoll et al., 2016). Furthermore, some pathogens are phagocytosed and delivered to lysosomes and subsequently withstand the hostile environment of the lysosomal pathway through the release of neutralizing factors (Voth and Heinzen, 2007).

Several pathogens use more than one of these strategies to circumvent host cell destruction; in fact, one of the most notorious pathogens, *Mycobacterium tuberculosis*, has evolved multiple mechanisms to avoid killing by immune cells as well as recognition by the adaptive immune system. For example, *M. tuberculosis* manipulates the host ability to detect and internalize the bacilli through pathogen-associated molecular patterns (PAMPs) (Stamm et al., 2015). Also, once inside the host cell, *M. tuberculosis* uses a range of strategies to avoid destruction, including manipulation of V-ATPase levels to avoid lysosomal acidification (Rohde et al., 2007), expression of genes that allow the bacilli to withstand the low pH of the endosomal/lysosomal pathway (Vandal et al., 2008), neutralizing reactive nitrogen and oxygen species (Shiloh and Nathan, 2000), resisting delivery to autophagosomes (Deretic, 2005; Romagnoli et al., 2012), counter-balancing iron depletion encountered upon phagocytosis (Weiss and Schaible, 2015) and interfering with lysosomal delivery following phagocytosis (Pieters, 2008; Gengenbacher and Kaufmann, 2012).

Inhibition of lysosomal delivery is an important trait of pathogenic mycobacteria, and the bacilli devote considerable efforts toward achieving that goal (Rohde et al., 2007; Pieters, 2008). This is achieved through the release of molecules, both signaling molecules, lipids and proteins (Cowley et al., 2004; Walburger et al., 2004; Hmama et al., 2015; Lovewell et al., 2016), as well as through the recruitment of host factors allowing mycobacterial escape from lysosomal degradation.

### Role for coronin 1 in the interaction of *M. tuberculosis* with macrophages

One of the host proteins co-opted by *M. tuberculosis* to avoid lysosomal killing is coronin 1 (encoded by the *coro1a* gene; for a discussion on the coronin nomenclature see; Pieters et al., 2013), a ~51 kD protein that is located in the macrophage cytosol and cell cortex. Coronin 1, also known as P57 or TACO, for Tryptophan Aspartate containing Coat protein, was identified in a search for host components potentially involved in the intracellular survival of mycobacteria within macrophages (Ferrari et al., 1999). Coronin 1 is a member of the highly conserved family of coronin proteins whose members are expressed across the eukaryotic kingdom and are characterized by the presence of a central WD (tryptophan-aspartate) 40 repeat that in coronin 1-folds in a 7-bladed beta propeller (Suzuki et al., 1995; Okumura et al., 1998; Gatfield et al., 2005; Appleton et al., 2006). Coronin 1 is highly expressed in all hematopoietic cells as well as to a lower degree in neurons (Ferrari et al., 1999; Nal et al., 2004; Jayachandran et al., 2014). Upon entry of pathogenic mycobacteria into macrophages, coronin 1 is retained on phagosomes containing viable, but not killed mycobacteria and its retention on phagosomes prevents intracellular killing of *M. tuberculosis* through activation of the Ca^2+^/calcineurin pathway (Jayachandran et al., 2007). Apart from *M. tuberculosis, M. leprae* as well as virulent *H. pylori* recruit coronin 1 to their intracellular niche, although the exact consequences of coronin 1 retention for pathogen survival in these latter cases remain unclear (Zheng and Jones, 2003; Suzuki et al., 2006).

## Coronins in dictyostelium

Coronin was initially isolated as a 55 kD actin/myosin-interacting molecule from *Dictyostelium discoideum* lysates (de Hostos et al., 1991). Subsequently, it was realized that *Dictyostelium* also expresses a so-called “tandem” coronin containing a duplicated tryptophan-aspartate repeat-containing region, now referred to as coronin B (Shina et al., 2010). Coronin B assembles with actin and deletion of this coronin in *Dictyostelium* was shown to both enhance as well as reduce phagocytosis, dependent on the particles to be internalized (Shina et al., 2010). Coronin B was proposed to act upstream of the suppressor of cAMP receptor (SCAR) and Wiskott-Aldrich syndrome protein (WASp) (Swaminathan et al., 2015) which connect signals from G protein-coupled receptors and cell surface tyrosine receptors to the actin cytoskeleton, respectively (Bear et al., 1998; Pollitt and Insall, 2009).

In *Dictyostelium*, coronin A is involved in a diverse array of activities, including cell motility, cAMP-mediated chemotaxis, and cytokinesis (de Hostos et al., 1993; Nagasaki et al., 2002). Given the initial isolation of coronin A with actin/myosin, the roles for coronin A in the above-mentioned activities have been attributed to the capacity of *Dictyostelium* coronin A in modulating the F-actin cytoskeleton. Coronin A is localized within regions of actin turnover (Maniak et al., 1995; Heinrich et al., 2008), leading to the conclusion that *Dictyostelium* coronin A is a regulator of the F-actin cytoskeleton, thereby modulating chemotaxis, cell motility and cytokinesis; it should however be noted that *Dictyostelium* lacking coronin A do not show an obvious alteration in the assembly or localization of actin filaments (de Hostos et al., 1993). Separate work using yeast coronin (Crn1) has shown that while deletion of yeast Crn1 does not result in an aberrant F-actin cytoskeleton (Heil-Chapdelaine et al., 1998; Goode et al., 1999), Crn1 binds to and bundles F-actin *in vitro* (Goode et al., 1999) as well as can modulate F-actin polymerization either positively (Liu et al., 2011) or negatively (Humphries et al., 2002) in an actin-related protein 2/3 (ARP2/3) complex-dependent manner. Indeed, yeast Crn1 possesses a number of regions of homology with actin- and tubulin-binding proteins, including microtubule- and F-actin/ARP2/3-interacting domains (Liu et al., 2011), that are lacking in most other coronins (Eckert et al., 2011).

More recent work that revisited the role for coronin A in *Dictyostelium* found that coronin A is important for the initiation of multicellular differentiation following deprivation of nutrients (Vinet et al., 2014); in *Dictyostelium*, nutrient starvation induces the aggregation of individual amoebae into a multicellular structure ultimately forming a fruiting body. Such aggregation is mediated by the second messenger cAMP, that functions both as a chemoattractant as well as an intracellular signal mediating gene transcription. Aggregation is initiated by cell density and nutrient-sensing factors released by the starving culture that induce the expression of genes involved in multicellular aggregation (Devreotes, 1989; Loomis, 2014). It was found that coronin A plays an essential role in the initiation of this developmental program by being involved in the response to factors secreted during the transition from growth to development of the cells. Since application of cAMP to coronin A-deficient cells is sufficient to restore chemotaxis and multicellular aggregation, coronin A appears to be dispensable for the cAMP relay as well as for processes downstream of cAMP. Also, the fact that folate chemotaxis occurs normally in the absence of coronin A argues against an exclusive role for coronin A in cytoskeletal remodeling (Vinet et al., 2014). Furthermore, consistent with earlier results showing that F-actin rearrangement is not required for the initiation of cAMP signaling (Parent et al., 1998; Kriebel et al., 2008), coronin A-dependent induction of genes required for development such as *aca* and *carA* does not require F-actin-based rearrangement (Vinet et al., 2014).

These data suggests that instead of modulating F-actin, coronin A is responsible for the sensing of factors secreted in the conditioned medium (Vinet et al., 2014). Coronin A may function downstream of conditioned medium factor (CMF) (Jain et al., 1992; Yuen et al., 1995), or possibly other, as yet undefined factors that are essential for the initiation of cAMP-dependent chemotaxis and aggregation. Whether the motility and cytokinesis defects observed upon coronin A deletion (de Hostos et al., 1993; Vinet et al., 2014) are related to a possible function of coronin A in the modulation of the cAMP pathway or linked to a role for coronin A in F-actin-mediated processes remains to be clarified. In this context, it is interesting to note that in *Dictyostelium*, myosin-independent cytokinesis (one of the two types of cytokinesis, the other one being myosin-II-dependent, see Nagasaki et al., 2002; Li, 2007) has been linked to both coronin A as well as AmiA (also known as Pianissimo, a target of rapamycin complex (TORC) 2-associated protein) that in *Dictyostelium* is a cytosolic regulator of adenylate cyclase (Pergolizzi et al., 2002). Furthermore, recent work implicated coronin A in regulating the availability of GTP-Rac through a domain with homology to a Cdc42- and Rac-interactive binding (CRIB) motif for activation of downstream effectors, thereby being responsible for myosin II disassembly (Swaminathan et al., 2014). Interestingly, this CRIB-like domain was found to be dispensable for the role of coronin A in cytokinesis, since expression of a mutant coronin A lacking Rac binding activity rescued the cytokinesis defect of coronin A-deficient cells (Swaminathan et al., 2014). This suggest that coronin A domains distinct from the CRIB-like motif are involved in the regulation of cytokinesis.

The precise role for *Dictyostelium* coronins in the modulation of bacterial uptake and survival is less clear. A role for coronin A in phagocytosis appears to be dependent on the type of cargo that is internalized; upon coronin A deletion, phagocytosis of *E. coli* and yeast particles is reduced, while uptake of *Mycobacterium marinum* is enhanced (Maniak et al., 1995; Solomon et al., 2003). Also, it should be noted that yeast phagocytosis is increased upon coronin B deletion (Shina et al., 2011). Furthermore, while coronin A becomes enriched on newly formed phagosomes, it is rapidly dissociated once the bacteria have been fully internalized (Maniak et al., 1995; Rauchenberger et al., 1997; Lu and Clarke, 2005; Hagedorn and Soldati, 2007). For both *M. marinum* as well as *Legionella pneumonia*, deletion of coronin A renders *Dictyostelium* more permissive for intracellular bacterial growth (Solomon et al., 2000, 2003). These data suggest that in *Dictyostelium* coronin A may play a protective role for the host, although the mechanisms involved remain unclear.

## Coronin 1 function in mammalian leukocytes

As described above, in resting macrophages, the only role for coronin 1 appears to be the modulation of the intracellular trafficking and survival of *M. tuberculosis* (Jayachandran et al., 2007, 2008). Given the proposed role for *Dictyostelium* coronin A in the modulation of F-actin-dependent processes such as chemotaxis and cytokinesis, it was initially anticipated that its mammalian homolog in macrophages, coronin 1, also modulates F-actin. However, in macrophages depleted for coronin 1 either by short interference (si)RNA or gene ablation, actin-dependent functions appear to be unperturbed as judged by the analysis of cell motility, macropinocytosis and membrane ruffling (Jayachandran et al., 2007, 2008). In contrast to studies using macrophages from coronin 1-deficient mice (Jayachandran et al., 2007) or J774 macrophages depleted for coronin 1 by siRNA (Jayachandran et al., 2008), TAT-mediated transduction of the WD repeat domain of coronin 1 in RAW 264.7 macrophages and neutrophils was shown to affect early phagocytosis (Yan et al., 2005, 2007); this may reflect differences in the experimental setup (such as time to allow uptake) or differential requirements for coronin 1 in phagocytosis in different macrophage and/or cell types; alternatively, especially given the propensity of coronin 1 mutants to cause aggregation, the introduction of misfolded protein domains may compromise cellular functions such as chemotaxis and phagocytosis (Gatfield et al., 2005). Whereas coronin 1 does not appear to be required for the functionality of resting macrophages, during an inflammatory stimulus coronin 1 is responsible for reprogramming the uptake pathway from phagocytosis to macropinocytosis in order to rapidly eliminate pathogens, a function that is dependent on activation of phosphoinositol (PI)-3-kinase (Bosedasgupta and Pieters, 2014). Furthermore, coronin 1 is largely dispensable for the functioning of B cells, mast cells, dendritic cells and natural killer cells, although the latter have been described to be affected by coronin 1 mutation in human (Moshous et al., 2013; Mace and Orange, 2014; Jayachandran and Pieters, 2015; Tchang et al., 2017). Also, coronin 1 was found to be dispensable for neutrophil function and recruitment in an *in vivo* model of liver injury and concanavanin A-induced hepatitis (Combaluzier and Pieters, 2009; Siegmund et al., 2013); in humans, coronin 1 has been associated with neutrophil survival and recent work has implicated coronin 1 in integrin-mediated functioning (Moriceau et al., 2009; Pick et al., 2017).

The in-depth analysis of coronin 1-deficient mice revealed that besides protecting intracellular mycobacteria from degradation within macrophages, a major function of coronin 1 is to regulate peripheral naïve T cell homeostasis; upon depletion of coronin 1, naïve T cells are virtually absent despite a normal development and selection in the thymus (Föger et al., 2006; Haraldsson et al., 2008; Mueller et al., 2008; Shiow et al., 2008; Lang et al., 2017). The role for coronin 1 in maintaining peripheral naïve T cells is conserved in humans: deletion or mutation of the *coro1a* gene has been reported to result in a selective depletion of naïve T cells (Moshous et al., 2013; Yee et al., 2016), or, when *coro1a* deletion or mutation is combined with other genetic aberrations, in more complex phenotypes including B and NK cell deficits besides the naïve T cell depletion (Shiow et al., 2009; Mace and Orange, 2014; Stray-Pedersen et al., 2014; Punwani et al., 2015; Yee et al., 2016). The mechanism underlying coronin 1-dependent naïve T cell survival remains controversial; in one study, coronin 1 was suggested to modulate the F-actin cytoskeleton, thereby regulating T cell survival (Föger et al., 2006); however, separate studies showed that altered F-actin levels do not correlate with T cell viability nor are other actin-dependent leukocyte functions affected by coronin 1 deletion (Jayachandran et al., 2007; Mueller et al., 2011). Instead, coronin 1-deficient T cells were shown to be unable to respond to a range of T cell stimuli and the defect was narrowed down to be at the level of activation of the Ca^2+^/calcineurin pathway (Haraldsson et al., 2008; Mueller et al., 2008).

Further analysis of mice lacking coronin 1 revealed an important function of this coronin family member in neurons, where it regulates Ca^2+^- and cAMP-dependent signaling thereby modulating various neuronal activities, including cognition and behavior as well as target innervation (Jayachandran et al., 2014; Suo et al., 2014). It is also interesting to note that *M. tuberculosis* is known to subvert host cAMP signaling (Agarwal et al., 2009), and retention of coronin 1 at the phagosomal membrane may be part of this strategy. Whether and how Ca^2+^ and cAMP signaling are interconnected and whether coronin 1-dependent cAMP signaling plays a role in T cell homeostasis and mycobacterial survival within macrophages remains to be analyzed.

## A concerted role for coronins in *Dictyostelium* and mammals?

The available information on the role for coronins in *Dictyostelium* and mammals suggests that these proteins play diverse roles in a number of physiological processes. The hallmark of all coronin protein family members is their central WD40 repeat, that folds into a beta propeller structure. Beta-propellers, also known as beta-transducin repeats, form structural domains that are involved in protein-protein interaction (Smith, 2008). Both in *Dictyostelium* as well as in mammalian cells, several coronin family members colocalize with and are associated with actin (de Hostos et al., 1991; Shina et al., 2010; Pieters et al., 2013) whereas for a number of mammalian coronin proteins (coronin 2, 5, and 7) actin binding remains unclear (see e.g., Rybakin et al., 2004; Cai et al., 2007). It of course remains possible, especially in mammals with up to 7 coronin molecules being expressed, that the role for coronins in actin rearrangement is redundant and therefore single deletions may not result in an actin-dependent phenotype. On the other hand, it could be that the interaction of coronin molecules with the actin cytoskeleton ensures a local source of coronin molecules to allow conversion of extracellular signals into local changes in the cortical actin cytoskeleton (Wang et al., 1998; Eichinger et al., 1999; Gatfield et al., 2005). Such a role for coronins is consistent not only with their sequence as well as structural homology with the beta subunit of trimeric G proteins that function downstream of G protein-coupled receptor molecules (de Hostos et al., 1991; Gatfield et al., 2005; Appleton et al., 2006), but also with the activities of *Dictyostelium* coronin A and mammalian coronin 1 in the modulation of Ca^2+^- and cAMP-dependent signal transduction pathways (Jayachandran et al., 2014; Suo et al., 2014; Vinet et al., 2014). Also, recent work linking coronins to the activation of small GTP binding proteins, that are well known regulators of the actin cytoskeleton (Berzat and Hall, 2010; Castro-Castro et al., 2011; Swaminathan et al., 2014), suggests that coronins may be placed upstream of F-actin reorganization. Interestingly, in both *Dictyostelium* as well as mammalian cells, the role for coronin in the activation of Ca^2+^/cAMP signaling could be separated from a potential involvement in F-actin rearrangement (Mueller et al., 2007, 2011; Jayachandran et al., 2014; Vinet et al., 2014). How, exactly, coronin molecules are being regulated is unknown. Also, the molecules upstream of coronin 1 possibly involved in the sensing of extracellular signals remain to be identified. In light of the here described roles for mammalian and *Dictyostelium* coronins in the trafficking and survival of intracellular pathogens, elucidation of these upstream receptor molecules may also shed light on the intricate relationship of pathogenic microbes and their eukaryotic hosts.

## Author contributions

All authors listed have made a substantial, direct and intellectual contribution to the work, and approved it for publication.

### Conflict of interest statement

The authors declare that the research was conducted in the absence of any commercial or financial relationships that could be construed as a potential conflict of interest.

## References

[B1] Aderem A. Underhill D. M. (1999). Mechanisms of phagocytosis in macrophages. Annu. Rev. Immunol. 17, 593–623. 10.1146/annurev.immunol.17.1.593 10358769

[B2] Agarwal N. Lamichhane G. Gupta R. Nolan S. Bishai W. R. (2009). Cyclic AMP intoxication of macrophages by a *Mycobacterium tuberculosis* adenylate cyclase. Nature 460, 98–102. 10.1038/nature08123 19516256

[B3] Allen L. A. Aderem A. (1996). Mechanisms of phagocytosis. Curr. Opin. Immunol. 8, 36–40. 10.1016/S0952-7915(96)80102-6 8729444

[B4] Appleton B. A. Wu P. Wiesmann C. (2006). The crystal structure of murine coronin-1: a regulator of actin cytoskeletal dynamics in lymphocytes. Structure 14, 87–96. 10.1016/j.str.2005.09.013 16407068

[B5] Bear J. E. Rawls J. F. Saxe C. L. III. (1998). SCAR, a WASP-related protein, isolated as a suppressor of receptor defects in late Dictyostelium development. J. Cell Biol. 142, 1325–1335. 10.1083/jcb.142.5.1325 9732292 PMC2149354

[B6] Berzat A. Hall A. (2010). Cellular responses to extracellular guidance cues. EMBO J. 29, 2734–2745. 10.1038/emboj.2010.170 20717143 PMC2924648

[B7] Blum J. S. Wearsch P. A. Cresswell P. (2013). Pathways of antigen processing. Annu. Rev. Immunol. 31, 443–473. 10.1146/annurev-immunol-032712-095910 23298205 PMC4026165

[B8] Bosedasgupta S. Pieters J. (2014). Inflammatory stimuli reprogram macrophage phagocytosis to macropinocytosis for the rapid elimination of pathogens. PLoS Pathog. 10:e1003879. 10.1371/journal.ppat.1003879 24497827 PMC3907376

[B9] Cai L. Marshall T. W. Uetrecht A. C. Schafer D. A. Bear J. E. (2007). Coronin 1B coordinates Arp2/3 complex and cofilin activities at the leading edge. Cell 128, 915–929. 10.1016/j.cell.2007.01.031 17350576 PMC2630706

[B10] Castro-Castro A. Ojeda V. Barreira M. Sauzeau V. Navarro-Lérida I. Muriel O.. (2011). Coronin 1A promotes a cytoskeletal-based feedback loop that facilitates Rac1 translocation and activation. EMBO J. 30, 3913-3927. 10.1038/emboj.2011.310 21873980 PMC3209784

[B11] Celli J. Gorvel J. P. (2004). Organelle robbery: brucella interactions with the endoplasmic reticulum. Curr. Opin. Microbiol. 7, 93–97. 10.1016/j.mib.2003.11.001 15036147

[B12] Combaluzier B. Pieters J. (2009). Chemotaxis and phagocytosis in neutrophils is independent of coronin 1. J. Immunol. 182, 2745–2752. 10.4049/jimmunol.0801812 19234169

[B13] Cowley S. Ko M. Pick N. Chow R. Downing K. J. Gordhan B. G.. (2004). The *Mycobacterium tuberculosis* protein serine/threonine kinase PknG is linked to cellular glutamate/glutamine levels and is important for growth *in vivo*. Mol. Microbiol. 52, 1691–1702. 10.1111/j.1365-2958.2004.04085.x 15186418

[B14] de Hostos E. L. Bradtke B. Lottspeich F. Guggenheim R. Gerisch G. (1991). Coronin, an actin binding protein of *Dictyostelium discoideum* localized to cell surface projections, has sequence similarities to G protein beta subunits. EMBO J. 10, 4097–4104. 1661669 10.1002/j.1460-2075.1991.tb04986.x PMC453159

[B15] de Hostos E. L. Rehfuess C. Bradtke B. Waddell D. R. Albrecht R. Murphy J.. (1993). Dictyostelium mutants lacking the cytoskeletal protein coronin are defective in cytokinesis and cell motility. J. Cell Biol. 120, 163–173. 10.1083/jcb.120.1.163 8380174 PMC2119478

[B16] Deretic V. (2005). Autophagy in innate and adaptive immunity. Trends Immunol. 26, 523–528. 10.1016/j.it.2005.08.003 16099218

[B17] Devreotes P. (1989). *Dictyostelium discoideum*: a model system for cell-cell interactions in development. Science 245, 1054–1058. 10.1126/science.2672337 2672337

[B18] Eckert C. Hammesfahr B. Kollmar M. (2011). A holistic phylogeny of the coronin gene family reveals an ancient origin of the tandem-coronin, defines a new subfamily, and predicts protein function. BMC Evol. Biol. 11:268. 10.1186/1471-2148-11-268 21943019 PMC3203266

[B19] Eichinger L. Lee S. S. Schleicher M. (1999). Dictyostelium as model system for studies of the actin cytoskeleton by molecular genetics. Microsc. Res. Tech. 47, 124–134. 10.1002/(SICI)1097-0029(19991015)47:2<124::AID-JEMT5>3.0.CO;2-8 10523791

[B20] Escoll P. Mondino S. Rolando M. Buchrieser C. (2016). Targeting of host organelles by pathogenic bacteria: a sophisticated subversion strategy. Nat. Rev. Microbiol. 14, 5–19. 10.1038/nrmicro.2015.1 26594043

[B21] Ferrari G. Langen H. Naito M. Pieters J. (1999). A coat protein on phagosomes involved in the intracellular survival of mycobacteria. Cell 97, 435–447. 10338208 10.1016/s0092-8674(00)80754-0

[B22] Flannagan R. S. Jaumouillé V. Grinstein S. (2012). The cell biology of phagocytosis. Annu. Rev. Pathol. 7, 61–98. 10.1146/annurev-pathol-011811-132445. 21910624

[B23] Föger N. Rangell L. Danilenko D. M. Chan A. C. (2006). Requirement for coronin 1 in T lymphocyte trafficking and cellular homeostasis. Science 313, 839–842. 10.1126/science.1130563 16902139

[B24] Gatfield J. Albrecht I. Zanolari B. Steinmetz M. O. Pieters J. (2005). Association of the leukocyte plasma membrane with the actin cytoskeleton through coiled coil-mediated trimeric coronin 1 molecules. Mol. Biol. Cell 16, 2786–2798. 10.1091/mbc.E05-01-0042 15800061 PMC1142424

[B25] Gengenbacher M. Kaufmann S. H. (2012). *Mycobacterium tuberculosis*: success through dormancy. FEMS Microbiol. Rev. 36, 514–532. 10.1111/j.1574-6976.2012.00331.x. 22320122 PMC3319523

[B26] Goode B. L. Wong J. J. Butty A. C. Peter M. McCormack A. L. Yates J. R.. (1999). Coronin promotes the rapid assembly and cross-linking of actin filaments and may link the actin and microtubule cytoskeletons in yeast. J. Cell Biol. 144, 83–98. 10.1083/jcb.144.1.83 9885246 PMC2148128

[B27] Hagedorn M. Soldati T. (2007). Flotillin and RacH modulate the intracellular immunity of Dictyostelium to *Mycobacterium marinum* infection. Cell. Microbiol. 9, 2716–2733. 10.1111/j.1462-5822.2007.00993.x 17587329

[B28] Hamon M. A. Ribet D. Stavru F. Cossart P. (2012). Listeriolysin O: the Swiss army knife of Listeria. Trends Microbiol. 20, 360–368. 10.1016/j.tim.2012.04.006 22652164

[B29] Haraldsson M. K. Louis-Dit-Sully C. A. Lawson B. R. Sternik G. Santiago-Raber M. L. Gascoigne N. R.. (2008). The lupus-related Lmb3 locus contains a disease-suppressing Coronin-1A gene mutation. Immunity 28, 40–51. 10.1016/j.immuni.2007.11.023 18199416 PMC2274909

[B30] Heil-Chapdelaine R. A. Tran N. K. Cooper J. A. (1998). The role of Saccharomyces cerevisiae coronin in the actin and microtubule cytoskeletons. Curr. Biol. 8, 1281–1284. 10.1016/S0960-9822(07)00539-8 9822583

[B31] Heinrich D. Youssef S. Schroth-Diez B. Engel U. Aydin D. Blümmel J.. (2008). Actin-cytoskeleton dynamics in non-monotonic cell spreading. Cell Adh. Migr. 2, 58–68. 10.4161/cam.2.2.6190 19262103 PMC2634985

[B32] Hmama Z. Peña-Díaz S. Joseph S. Av-Gay Y. (2015). Immunoevasion and immunosuppression of the macrophage by *Mycobacterium tuberculosis*. Immunol. Rev. 264, 220–232. 10.1111/imr.12268 25703562

[B33] Humphries C. L. Balcer H. I. D'Agostino J. L. Winsor B. Drubin D. G. Barnes G.. (2002). Direct regulation of Arp2/3 complex activity and function by the actin binding protein coronin. J. Cell Biol. 159, 993–1004. 10.1083/jcb.200206113 12499356 PMC2173993

[B34] Jain R. Yuen I. S. Taphouse C. R. Gomer R. H. (1992). A density-sensing factor controls development in Dictyostelium. Genes Dev. 6, 390–400. 10.1101/gad.6.3.390 1547939

[B35] Jayachandran R. Gatfield J. Massner J. Albrecht I. Zanolari B. Pieters J. (2008). RNA interference in J774 macrophages reveals a role for coronin 1 in mycobacterial trafficking but not in actin-dependent processes. Mol. Biol. Cell 19, 1241–1251. 10.1091/mbc.E07-07-0640 18162581 PMC2262969

[B36] Jayachandran R. Liu X. Bosedasgupta S. Müller P. Zhang C. L. Moshous D.. (2014). Coronin 1 regulates cognition and behavior through modulation of cAMP/protein kinase A signaling. PLoS Biol. 12:e1001820. 10.1371/journal.pbio.1001820 24667537 PMC3965382

[B37] Jayachandran R. Pieters J. (2015). Regulation of immune cell homeostasis and function by coronin 1. Int. Immunopharmacol. 28, 825–828. 10.1016/j.intimp.2015.03.045. 25882105

[B38] Jayachandran R. Sundaramurthy V. Combaluzier B. Mueller P. Korf H. Huygen K.. (2007). Survival of mycobacteria in macrophages is mediated by coronin 1-dependent activation of calcineurin. Cell 130, 37–50. 10.1016/j.cell.2007.04.043 17632055

[B39] Kriebel P. W. Barr V. A. Rericha E. C. Zhang G. Parent C. A. (2008). Collective cell migration requires vesicular trafficking for chemoattractant delivery at the trailing edge. J. Cell Biol. 183, 949–961. 10.1083/jcb.200808105 19047467 PMC2592838

[B40] Lang M. J. Mori M. Ruer-Laventie J. Pieters J. (2017). A coronin 1-dependent decision switch in juvenile mice determines the population of the peripheral naive T cell compartment. J. Immunol. 199, 2421–2431. 10.4049/jimmunol.1700438 28821585

[B41] Li R. (2007). Cytokinesis in development and disease: variations on a common theme. Cell. Mol. Life Sci. 64, 3044–3058. 10.1007/s00018-007-7285-6 17882379 PMC11136365

[B42] Liu S. L. Needham K. M. May J. R. Nolen B. J. (2011). Mechanism of a concentration-dependent switch between activation and inhibition of Arp2/3 complex by coronin. J. Biol. Chem. 286, 17039–17046. 10.1074/jbc.M111.219964 21454476 PMC3089548

[B43] Loomis W. F. (2014). Cell signaling during development of Dictyostelium. Dev. Biol. 391, 1–16. 10.1016/j.ydbio.2014.04.001 24726820 PMC4075484

[B44] Lovewell R. R. Sassetti C. M. VanderVen B. C. (2016). Chewing the fat: lipid metabolism and homeostasis during *M. tuberculosis* infection. Curr. Opin. Microbiol. 29, 30–36. 10.1016/j.mib.2015.10.002 26544033

[B45] Lu H. Clarke M. (2005). Dynamic properties of Legionella-containing phagosomes in Dictyostelium amoebae. Cell. Microbiol. 7, 995–1007. 10.1111/j.1462-5822.2005.00528.x 15953031

[B46] Mace E. M. Orange J. S. (2014). Lytic immune synapse function requires filamentous actin deconstruction by Coronin 1A. Proc. Natl. Acad. Sci. U.S.A. 111, 6708–6713. 10.1073/pnas.1314975111 24760828 PMC4020046

[B47] Maniak M. Rauchenberger R. Albrecht R. Murphy J. Gerisch G. (1995). Coronin involved in phagocytosis: dynamics of particle-induced relocalization visualized by a green fluorescent protein Tag. Cell 83, 915–924. 10.1016/0092-8674(95)90207-4 8521515

[B48] Mellouk N. Enninga J. (2016). Cytosolic access of intracellular bacterial pathogens: the shigella paradigm. Front. Cell. Infect. Microbiol. 6:35. 10.3389/fcimb.2016.00035 27092296 PMC4820437

[B49] Moriceau S. Kantari C. Mocek J. Davezac N. Gabillet J. Guerrera I. C.. (2009). Coronin-1 is associated with neutrophil survival and is cleaved during apoptosis: potential implication in neutrophils from cystic fibrosis patients. J. Immunol. 182, 7254–7263. 10.4049/jimmunol.0803312 19454722

[B50] Moshous D. Martin E. Carpentier W. Lim A. Callebaut I. Canioni D.. (2013). Whole-exome sequencing identifies Coronin-1A deficiency in 3 siblings with immunodeficiency and EBV-associated B-cell lymphoproliferation. J. Allergy Clin. Immunol. 131, 1594–1603. 10.1016/j.jaci.2013.01.042 23522482 PMC3824285

[B51] Mueller P. Liu X. Pieters J. (2011). Migration and homeostasis of naive T cells depends on coronin 1-mediated prosurvival signals and not on coronin 1-dependent filamentous actin modulation. J. Immunol. 186, 4039–4050. 10.4049/jimmunol.1003352 21339362

[B52] Mueller P. Massner J. Jayachandran R. Combaluzier B. Albrecht I. Gatfield J.. (2008). Regulation of T cell survival through coronin-1-mediated generation of inositol-1,4,5-trisphosphate and calcium mobilization after T cell receptor triggering. Nat. Immunol. 9, 424–431. 10.1038/ni1570 18345003

[B53] Mueller P. Quintana A. Griesemer D. Hoth M. Pieters J. (2007). Disruption of the cortical actin cytoskeleton does not affect store operated Ca^2+^ channels in human T-cells. FEBS Lett. 581, 3557–3562. 10.1016/j.febslet.2007.06.068 17624329

[B54] Nagasaki A. de Hostos E. L. Uyeda T. Q. (2002). Genetic and morphological evidence for two parallel pathways of cell-cycle-coupled cytokinesis in Dictyostelium. J. Cell Sci. 115(Pt 10), 2241–2251. 11973364 10.1242/jcs.115.10.2241

[B55] Nal B. Carroll P. Mohr E. Verthuy C. Da Silva M. I. Gayet O.. (2004). Coronin-1 expression in T lymphocytes: insights into protein function during T cell development and activation. Int. Immunol. 16, 231–240. 10.1093/intimm/dxh022 14734608

[B56] Okumura M. Kung C. Wong S. Rodgers M. Thomas M. L. (1998). Definition of family of coronin-related proteins conserved between humans and mice: close genetic linkage between coronin-2 and CD45-associated protein. DNA Cell Biol. 17, 779–787. 10.1089/dna.1998.17.779 9778037

[B57] Parent C. A. Blacklock B. J. Froehlich W. M. Murphy D. B. Devreotes P. N. (1998). G protein signaling events are activated at the leading edge of chemotactic cells. Cell 95, 81–91. 9778249 10.1016/s0092-8674(00)81784-5

[B58] Pergolizzi B. Peracino B. Silverman J. Ceccarelli A. Noegel A. Devreotes P.. (2002). Temperature-sensitive inhibition of development in Dictyostelium due to a point mutation in the piaA gene. Dev. Biol. 251, 18–26. 10.1006/dbio.2002.0809 12413895

[B59] Pick R. Begandt D. Stocker T. J. Salvermoser M. Thome S. Böttcher R. T.. (2017). Coronin 1A, a novel player in integrin biology, controls neutrophil trafficking in innate immunity. Blood 130, 847–858. 10.1182/blood-2016-11-749622 28615221

[B60] Pieters J. (2000). MHC class II-restricted antigen processing and presentation. Adv. Immunol. 75, 159–208. 10.1016/S0065-2776(00)75004-8 10879284

[B61] Pieters J. (2008). *Mycobacterium tuberculosis* and the macrophage: maintaining a balance. Cell Host Microbe 3, 399–407. 10.1016/j.chom.2008.05.006 18541216

[B62] Pieters J. Müller P. Jayachandran R. (2013). On guard: coronin proteins in innate and adaptive immunity. Nat. Rev. Immunol. 13, 510–518. 10.1038/nri3465 23765056

[B63] Pollitt A. Y. Insall R. H. (2009). WASP and SCAR/WAVE proteins: the drivers of actin assembly. J. Cell Sci. 122(Pt 15), 2575–2578. 10.1242/jcs.023879 19625501 PMC2954249

[B64] Punwani D. Pelz B. Yu J. Arva N. C. Schafernak K. Kondratowicz K.. (2015). Coronin-1A: immune deficiency in humans and mice. J. Clin. Immunol. 35, 100–107. 10.1007/s10875-015-0130-z. 25666293 PMC4489527

[B65] Rauchenberger R. Hacker U. Murphy J. Niewöhner J. Maniak M. (1997). Coronin and vacuolin identify consecutive stages of a late, actin- coated endocytic compartment in Dictyostelium. Curr. Biol. 7, 215–218. 10.1016/S0960-9822(97)70093-9 9276759

[B66] Rohde K. Yates R. M. Purdy G. E. Russell D. G. (2007). *Mycobacterium tuberculosis* and the environment within the phagosome. Immunol. Rev. 219, 37–54. 10.1111/j.1600-065X.2007.00547.x 17850480

[B67] Romagnoli A. Etna M. P. Giacomini E. Pardini M. Remoli M. E. Corazzari M.. (2012). ESX-1 dependent impairment of autophagic flux by *Mycobacterium tuberculosis* in human dendritic cells. Autophagy 8, 1357–1370. 10.4161/auto.20881 22885411 PMC3442882

[B68] Rybakin V. Stumpf M. Schulze A. Majoul I. V. Noegel A. A. Hasse A. (2004). Coronin 7, the mammalian POD-1 homologue, localizes to the Golgi apparatus. FEBS Lett. 573, 161–167. 10.1016/j.febslet.2004.07.066 15327992

[B69] Sarantis H. Grinstein S. (2012). Subversion of phagocytosis for pathogen survival. Cell Host Microbe 12, 419–431. 10.1016/j.chom.2012.09.001 23084912

[B70] Shiloh M. U. Nathan C. F. (2000). Reactive nitrogen intermediates and the pathogenesis of Salmonella and mycobacteria. Curr. Opin. Microbiol. 3, 35–42. 10.1016/S1369-5274(99)00048-X 10679417

[B71] Shina M. C. Müller-Taubenberger A. Unal C. Schleicher M. Steinert M. Eichinger L.. (2011). Redundant and unique roles of coronin proteins in Dictyostelium. Cell. Mol. Life Sci. CMLS 68, 303–313. 10.1007/s00018-010-0455-y 20640912 PMC11114531

[B72] Shina M. C. Unal C. Eichinger L. Muller-Taubenberger A. Schleicher M. Steinert M.. (2010). A Coronin7 homolog with functions in actin-driven processes. J. Biol. Chem. 285, 9249–9261. 10.1074/jbc.M109.083725 20071332 PMC2838343

[B73] Shiow L. R. Paris K. Akana M. C. Cyster J. G. Sorensen R. U. Puck J. M. (2009). Severe combined immunodeficiency (SCID) and attention deficit hyperactivity disorder (ADHD) associated with a Coronin-1A mutation and a chromosome 16p11.2 deletion. Clin. Immunol. 131, 24–30. 10.1016/j.clim.2008.11.002 19097825 PMC2692687

[B74] Shiow L. R. Roadcap D. W. Paris K. Watson S. R. Grigorova I. L. Lebet T.. (2008). The actin regulator coronin 1A is mutant in a thymic egress-deficient mouse strain and in a patient with severe combined immunodeficiency. Nat. Immunol. 9, 1307–1315. 10.1038/ni.1662 18836449 PMC2672406

[B75] Siegmund K. Lee W. Y. Tchang V. S. Stiess M. Terracciano L. Kubes P.. (2013). Coronin 1 is dispensable for leukocyte recruitment and liver injury in concanavalin A-induced hepatitis. Immunol. Lett. 153, 62–70. 10.1016/j.imlet.2013.06.005 23856257

[B76] Smith T. F. (2008). Diversity of WD-repeat proteins. Subcell. Biochem. 48, 20–30. 10.1007/978-0-387-09595-0_3. 18925368

[B77] Solomon J. M. Leung G. S. Isberg R. R. (2003). Intracellular replication of *Mycobacterium marinum* within *Dictyostelium discoideum*: efficient replication in the absence of host coronin. Infect. Immun. 71, 3578–3586. 10.1128/IAI.71.6.3578-3586.2003 12761143 PMC155778

[B78] Solomon J. M. Rupper A. Cardelli J. A. Isberg R. R. (2000). Intracellular growth of Legionella pneumophila in *Dictyostelium discoideum*, a system for genetic analysis of host-pathogen interactions. Infect. Immun. 68, 2939–2947. 10.1128/IAI.68.5.2939-2947.2000 10768992 PMC97507

[B79] Stamm C. E. Collins A. C. Shiloh M. U. (2015). Sensing of *Mycobacterium tuberculosis* and consequences to both host and bacillus. Immunol. Rev. 264, 204–219. 10.1111/imr.12263 25703561 PMC4339209

[B80] Stray-Pedersen A. Jouanguy E. Crequer A. Bertuch A. A. Brown B. S. Jhangiani S. N.. (2014). Compound heterozygous CORO1A mutations in siblings with a mucocutaneous-immunodeficiency syndrome of epidermodysplasia verruciformis-HPV, molluscum contagiosum and granulomatous tuberculoid leprosy. J. Clin. Immunol. 34, 871–890. 10.1007/s10875-014-0074-8 25073507 PMC4386834

[B81] Suo D. Park J. Harrington A. W. Zweifel L. S. Mihalas S. Deppmann C. D. (2014). Coronin-1 is a neurotrophin endosomal effector that is required for developmental competition for survival. Nat. Neurosci. 17, 36–45. 10.1038/nn.3593 24270184 PMC3962792

[B82] Suzuki K. Nishihata J. Arai Y. Honma N. Yamamoto K. Irimura T.. (1995). Molecular cloning of a novel actin-binding protein, p57, with a WD repeat and a leucine zipper motif. FEBS Lett. 364, 283–288. 10.1016/0014-5793(95)00393-N 7758584

[B83] Suzuki K. Takeshita F. Nakata N. Ishii N. Makino M. (2006). Localization of CORO1A in the macrophages containing Mycobacterium leprae. Acta Histochem. Cytochem. 39, 107–112. 10.1267/ahc.06010 17327897 PMC1698865

[B84] Swaminathan K. Müller-Taubenberger A. Faix J. Rivero F. Noegel A. A. (2014). A Cdc42- and Rac-interactive binding (CRIB) domain mediates functions of coronin. Proc. Natl. Acad. Sci. U.S.A. 111, E25–E33. 10.1073/pnas.1315368111 24347642 PMC3890859

[B85] Swaminathan K. Stumpf M. Müller R. Horn A. C. Schmidbauer J. Eichinger L.. (2015). Coronin7 regulates WASP and SCAR through CRIB mediated interaction with Rac proteins. Sci. Rep. 5:14437. 10.1038/srep14437 26411260 PMC4585930

[B86] Tchang V. S. Y. Stiess M. Siegmund K. Karrer U. Pieters J. (2017). Role for coronin 1 in mouse NK cell function. Immunobiology 222, 291–300. 10.1016/j.imbio.2016.09.011 27717523

[B87] Vandal O. H. Pierini L. M. Schnappinger D. Nathan C. F. Ehrt S. (2008). A membrane protein preserves intrabacterial pH in intraphagosomal *Mycobacterium tuberculosis*. Nat. Med. 14, 849–854. 10.1038/nm.1795 18641659 PMC2538620

[B88] Vinet A. F. Fiedler T. Studer V. Froquet R. Dardel A. Cosson P.. (2014). Initiation of multicellular differentiation in *Dictyostelium discoideum* is regulated by coronin A. Mol. Biol. Cell 25, 688–701. 10.1091/mbc.E13-04-0219 24403600 PMC3937094

[B89] Voth D. E. Heinzen R. A. (2007). Lounging in a lysosome: the intracellular lifestyle of Coxiella burnetii. Cell. Microbiol. 9, 829–840. 10.1111/j.1462-5822.2007.00901.x. 17381428

[B90] Walburger A. Koul A. Ferrari G. Nguyen L. Prescianotto-Baschong C. Huygen K.. (2004). Protein kinase G from pathogenic mycobacteria promotes survival within macrophages. Science 304, 1800–1804. 10.1126/science.1099384 15155913

[B91] Wang Y. Liu J. Segall J. E. (1998). MAP kinase function in amoeboid chemotaxis. J. Cell Sci. 111 (Pt 3), 373–383. 9427685 10.1242/jcs.111.3.373

[B92] Weiss G. Schaible U. E. (2015). Macrophage defense mechanisms against intracellular bacteria. Immunol. Rev. 264, 182–203. 10.1111/imr.12266 25703560 PMC4368383

[B93] Yan M. Collins R. F. Grinstein S. Trimble W. S. (2005). Coronin-1 function is required for phagosome formation. Mol. Biol. Cell 16, 3077–3087. 10.1091/mbc.E04-11-0989 15829569 PMC1165393

[B94] Yan M. Di Ciano-Oliveira C. Grinstein S. Trimble W. S. (2007). Coronin function is required for chemotaxis and phagocytosis in human neutrophils. J. Immunol. 178, 5769–5778. 10.4049/jimmunol.178.9.5769 17442961

[B95] Yee C. S. Massaad M. J. Bainter W. Ohsumi T. K. Föger N. Chan A. C.. (2016). Recurrent viral infections associated with a homozygous CORO1A mutation that disrupts oligomerization and cytoskeletal association. J. Allergy Clin. Immunol. 137, 879-888.e872. 10.1016/j.jaci.2015.08.020 26476480 PMC4783242

[B96] Yuen I. S. Jain R. Bishop J. D. Lindsey D. F. Deery W. J. Van Haastert P. J.. (1995). A density-sensing factor regulates signal transduction in Dictyostelium. J. Cell Biol. 129, 1251–1262. 10.1083/jcb.129.5.1251 7775572 PMC2120463

[B97] Zheng P. Y. Jones N. L. (2003). Helicobacter pylori strains expressing the vacuolating cytotoxin interrupt phagosome maturation in macrophages by recruiting and retaining TACO (coronin 1) protein. Cell. Microbiol. 5, 25–40. 10.1046/j.1462-5822.2003.00250.x 12542468

